# Supplementary figures and images for: Structure-based electron-confurcation mechanism of the Ldh-EtfAB complex
Source: eLife. 2022 Jun 24;11:e77095. doi: 10.7554/eLife.77095 (PMC9232219; doi:10.7554/eLife.77095)

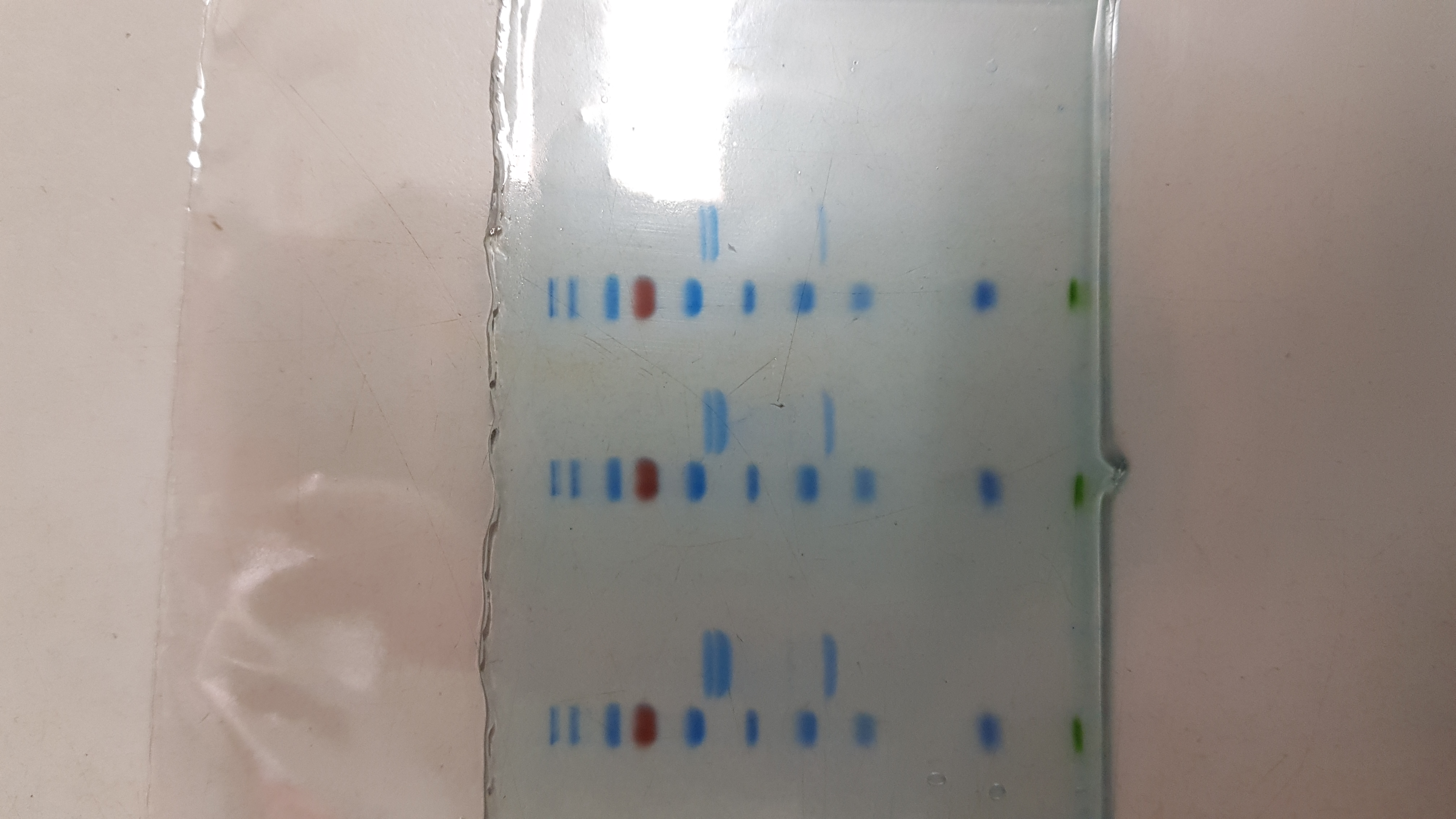

Supplement: Figure 2—source data 1. [file elife-77095-fig2-data1.zip › source_fig1/Fig. 1 -source1.jpg]

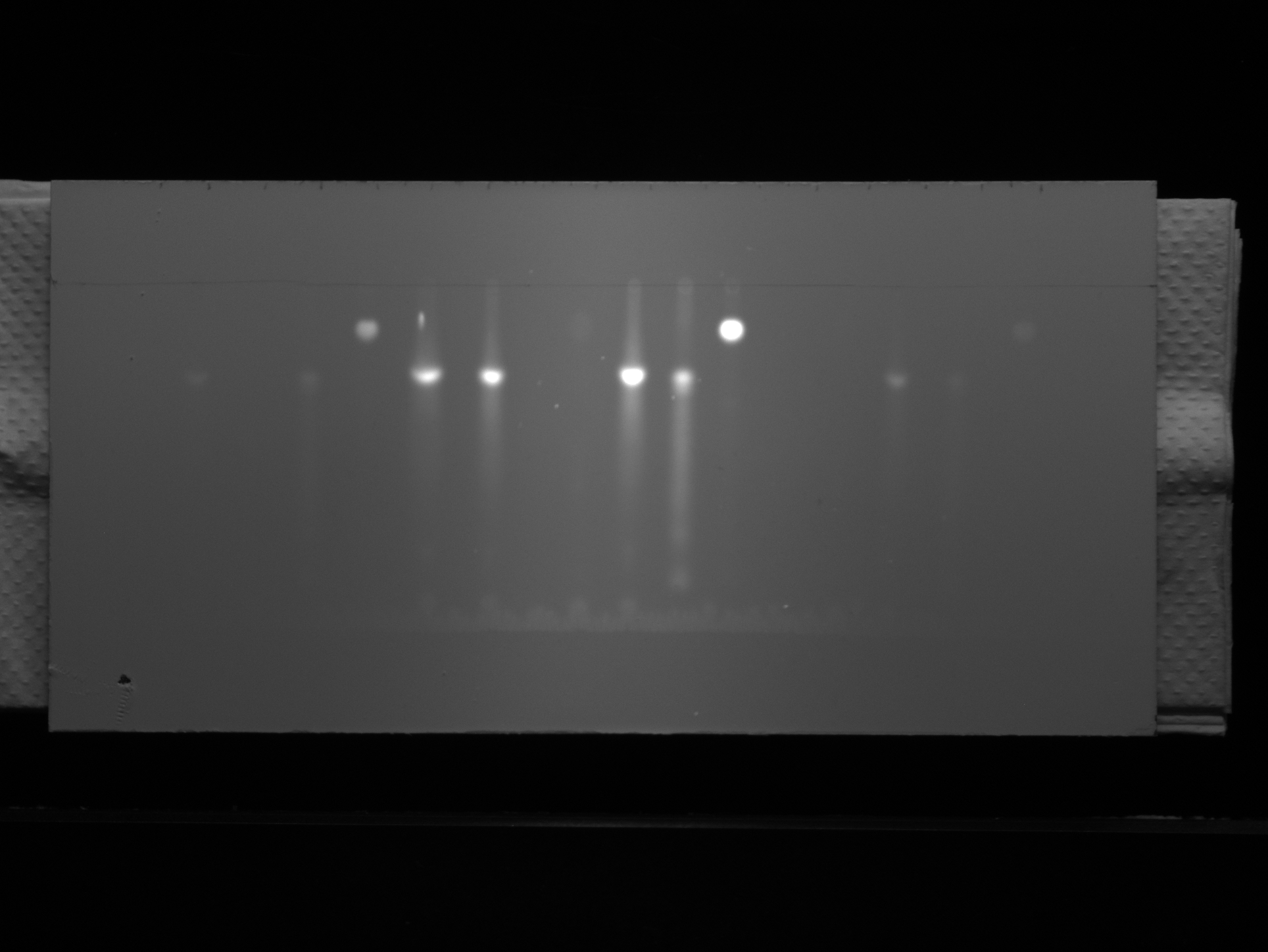

Supplement: Figure 2—source data 1. [file elife-77095-fig2-data1.zip › source_fig1/Fig. 1 -source2.tif]

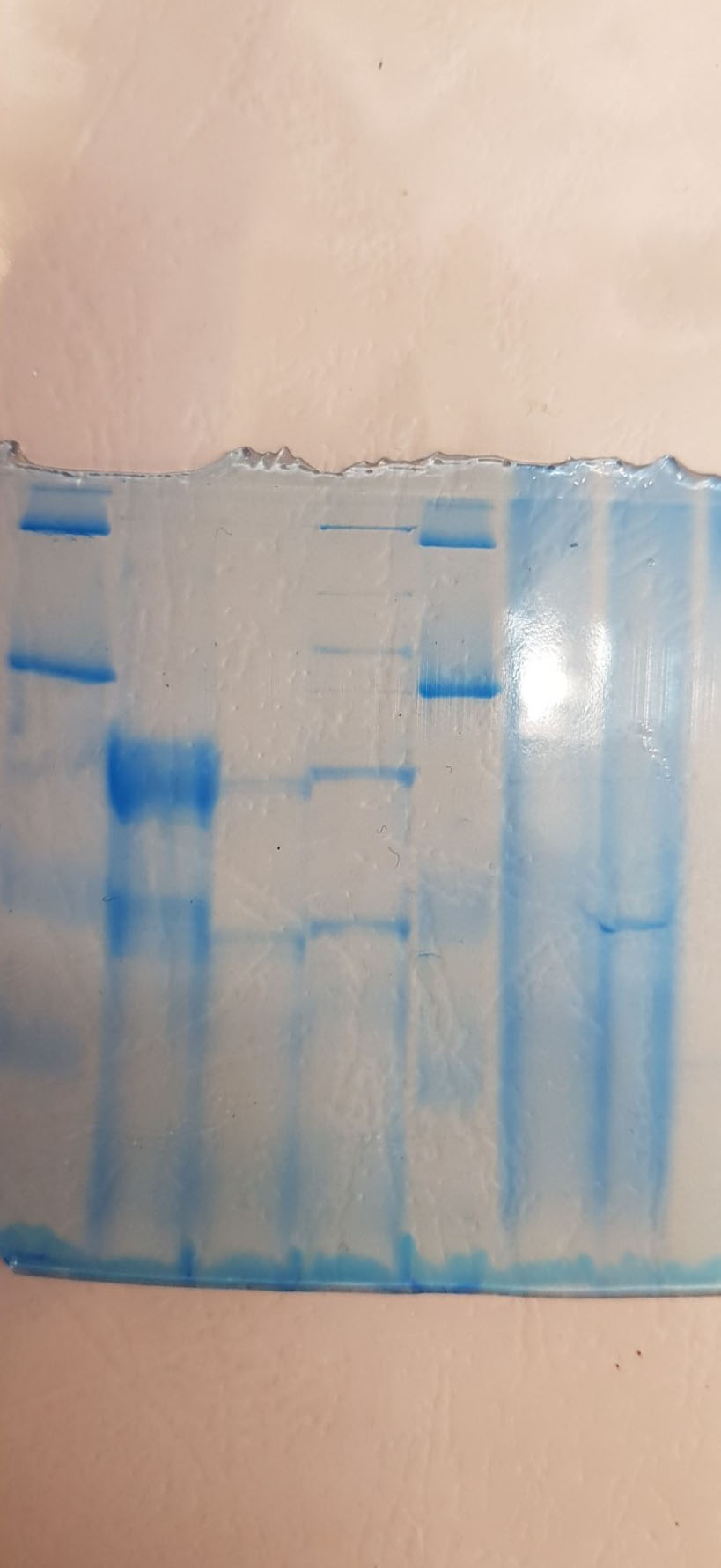

Supplement: Figure 2—source data 1. [file elife-77095-fig2-data1.zip › source_fig1/Fig. 1 -source3.jpg]

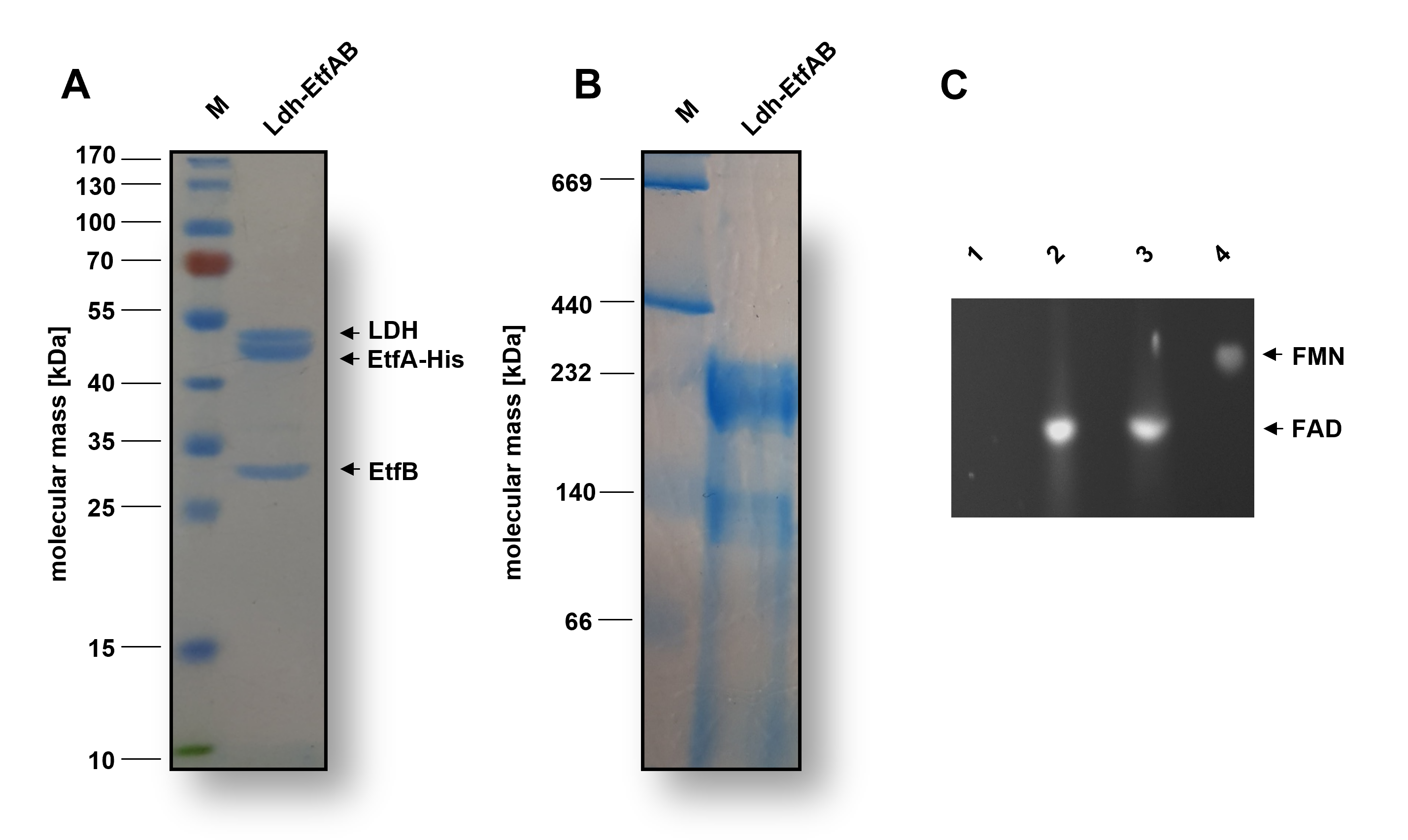

Supplement: Figure 2—source data 1. [file elife-77095-fig2-data1.zip › source_fig1/Fig. 1.tif]

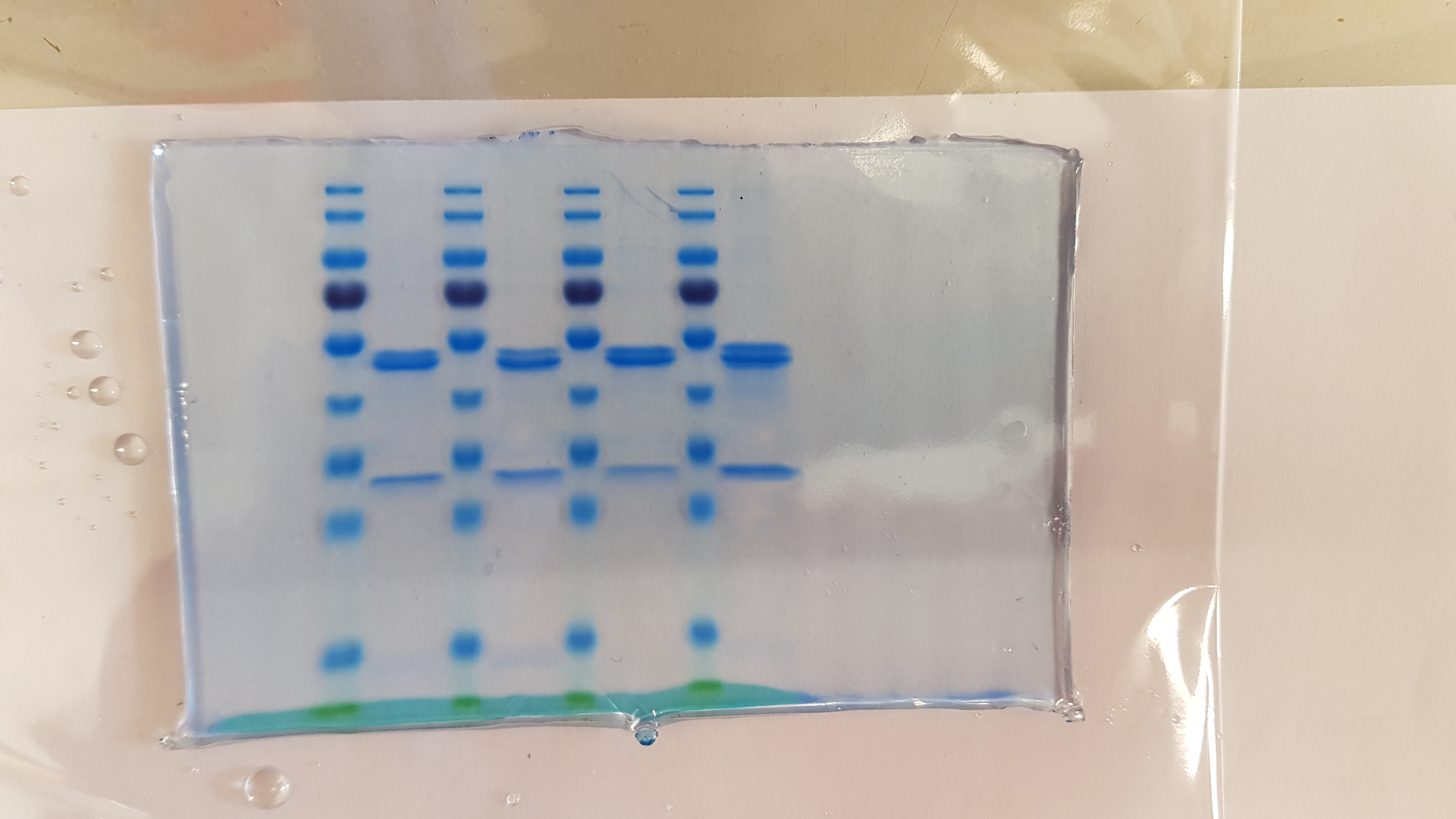

Supplement: Figure 7—figure supplement 1—source data 1. [file elife-77095-fig7-figsupp1-data1.zip › source_tab1supp/Fig. S5 supplement -source1.jpg]

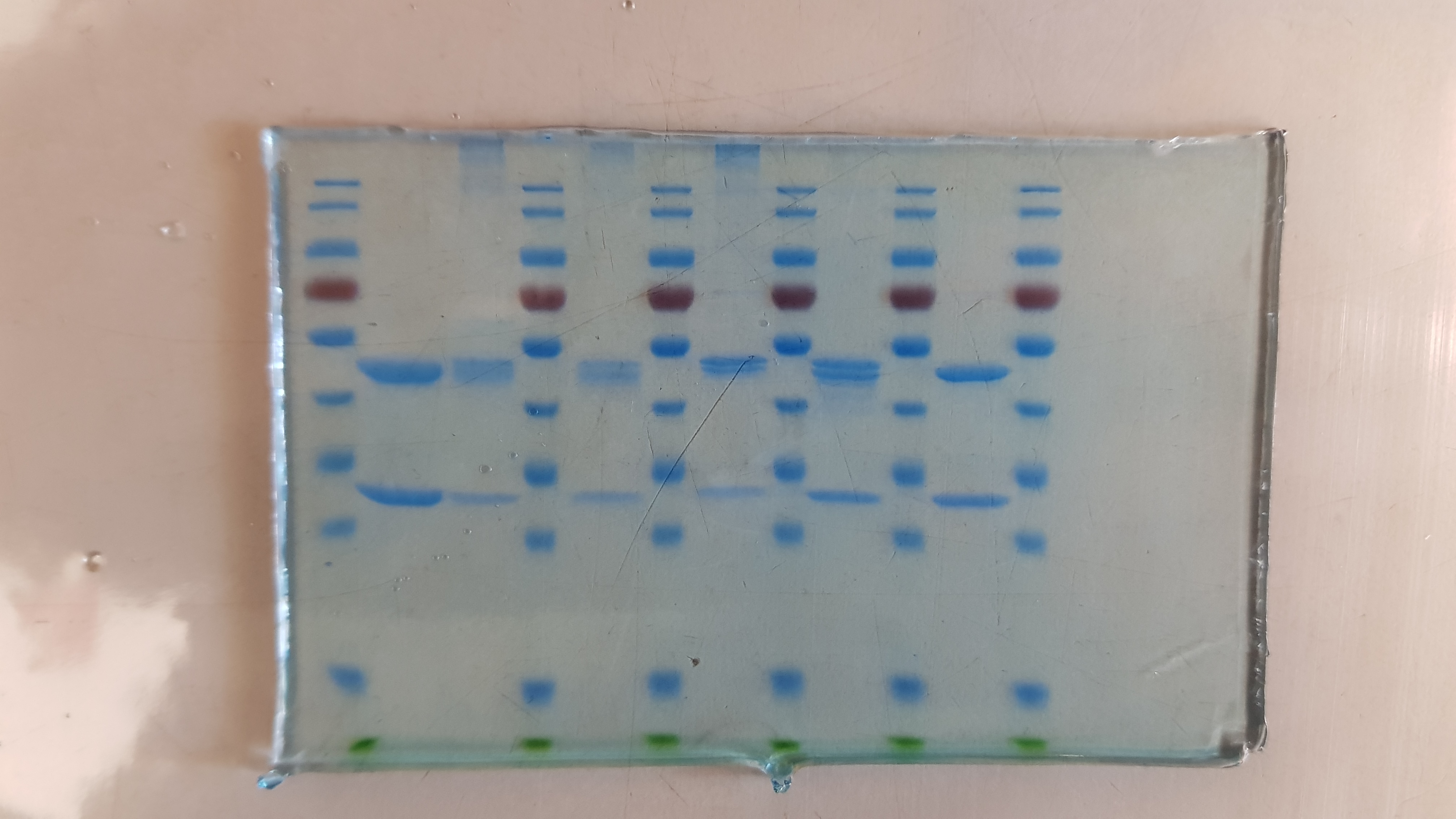

Supplement: Figure 7—figure supplement 1—source data 1. [file elife-77095-fig7-figsupp1-data1.zip › source_tab1supp/Fig. S5 supplement -source2.jpg]

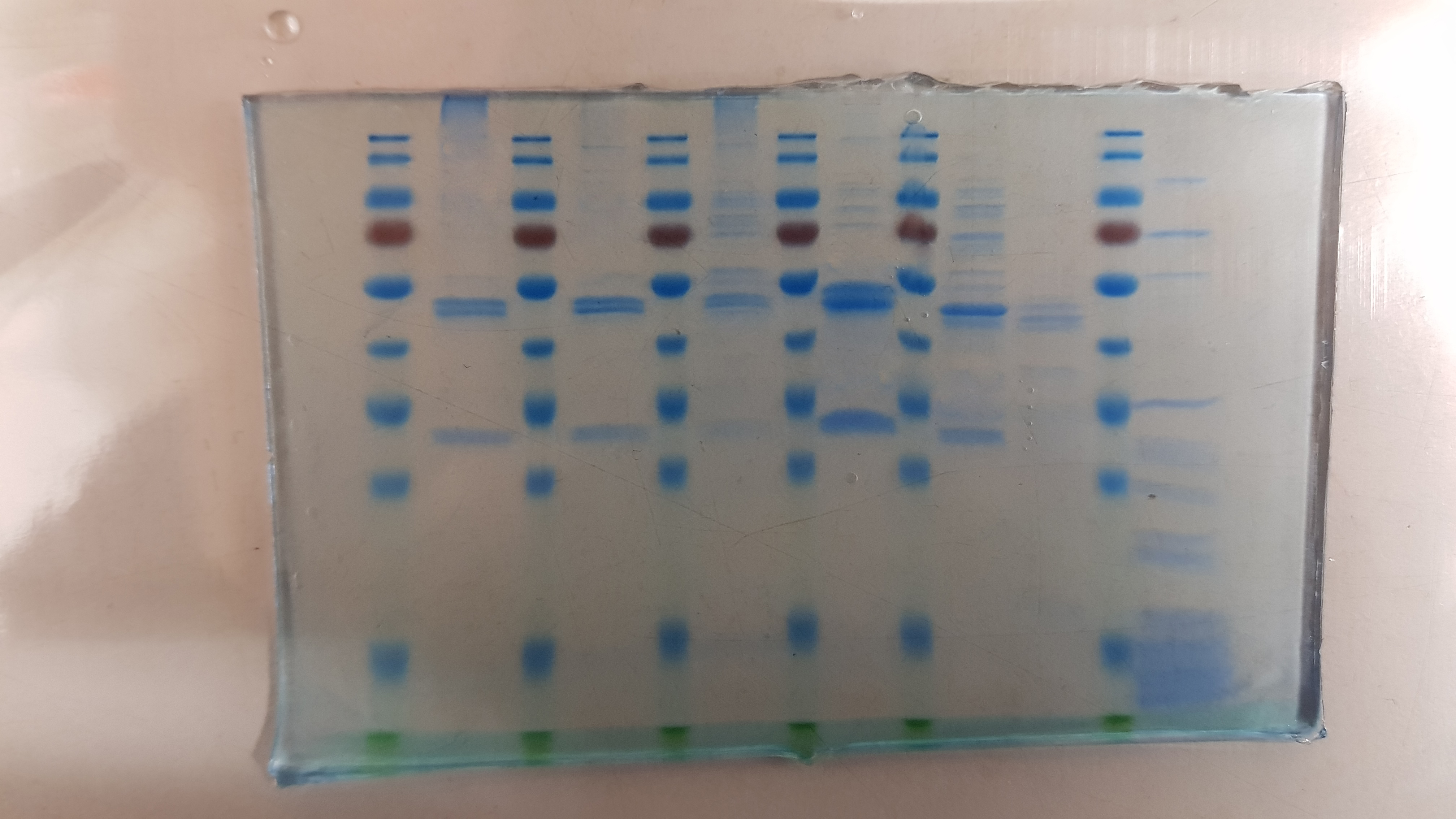

Supplement: Figure 7—figure supplement 1—source data 1. [file elife-77095-fig7-figsupp1-data1.zip › source_tab1supp/Fig. S5 supplement -source3.jpg]

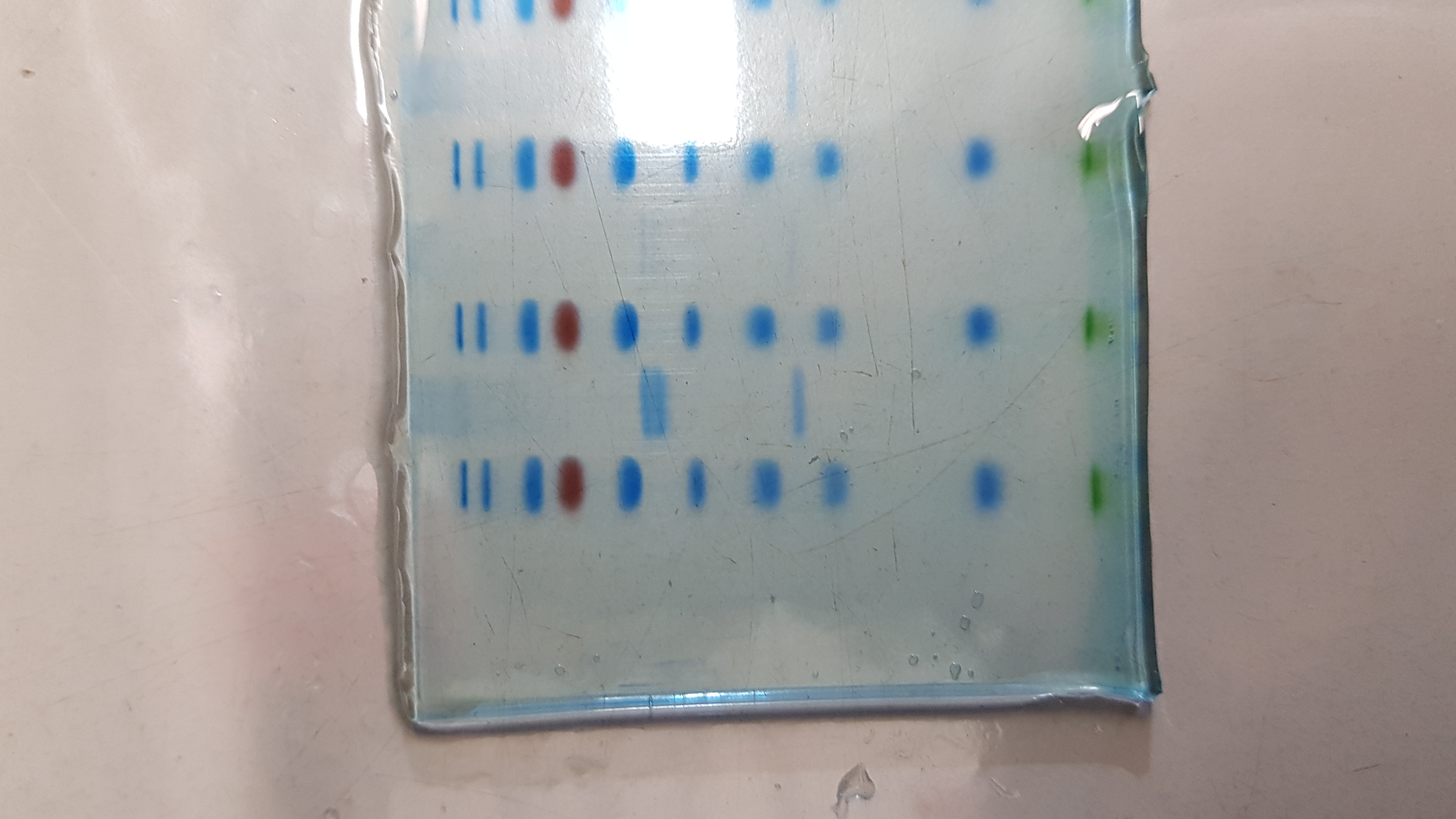

Supplement: Figure 7—figure supplement 1—source data 1. [file elife-77095-fig7-figsupp1-data1.zip › source_tab1supp/Fig. S5 supplement -source4.jpg]

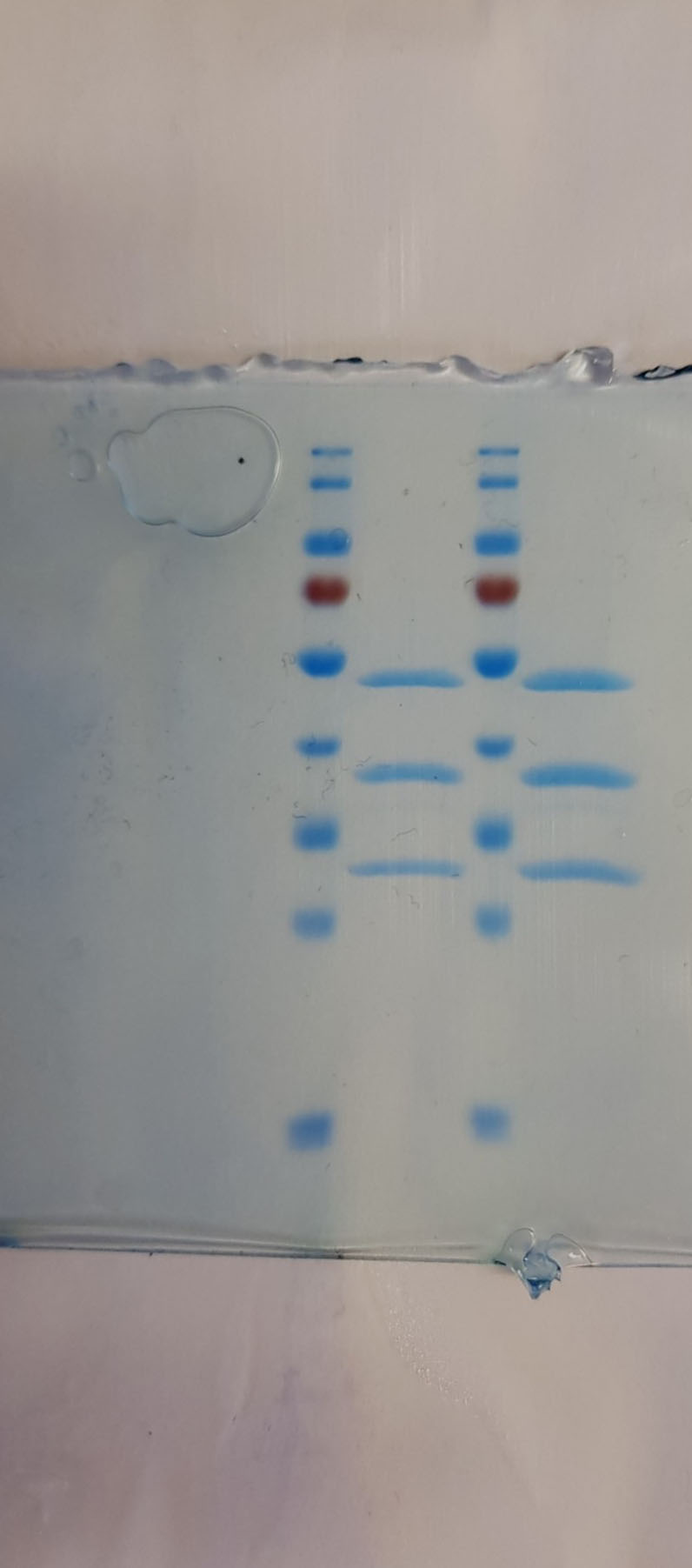

Supplement: Figure 7—figure supplement 1—source data 1. [file elife-77095-fig7-figsupp1-data1.zip › source_tab1supp/Fig. S5 supplement -source5.jpg]

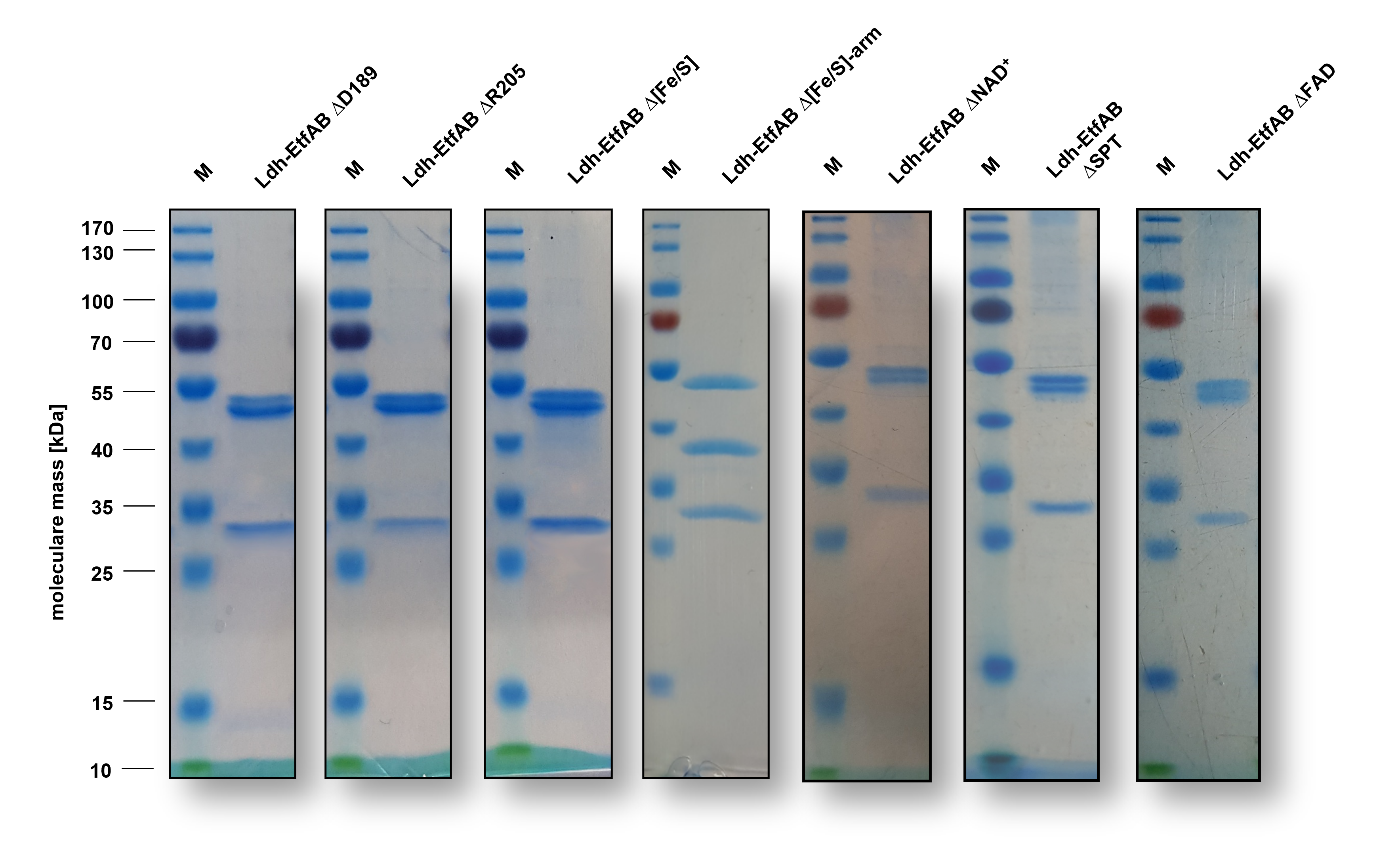

Supplement: Figure 7—figure supplement 1—source data 1. [file elife-77095-fig7-figsupp1-data1.zip › source_tab1supp/Fig. S5 supplement.tif]

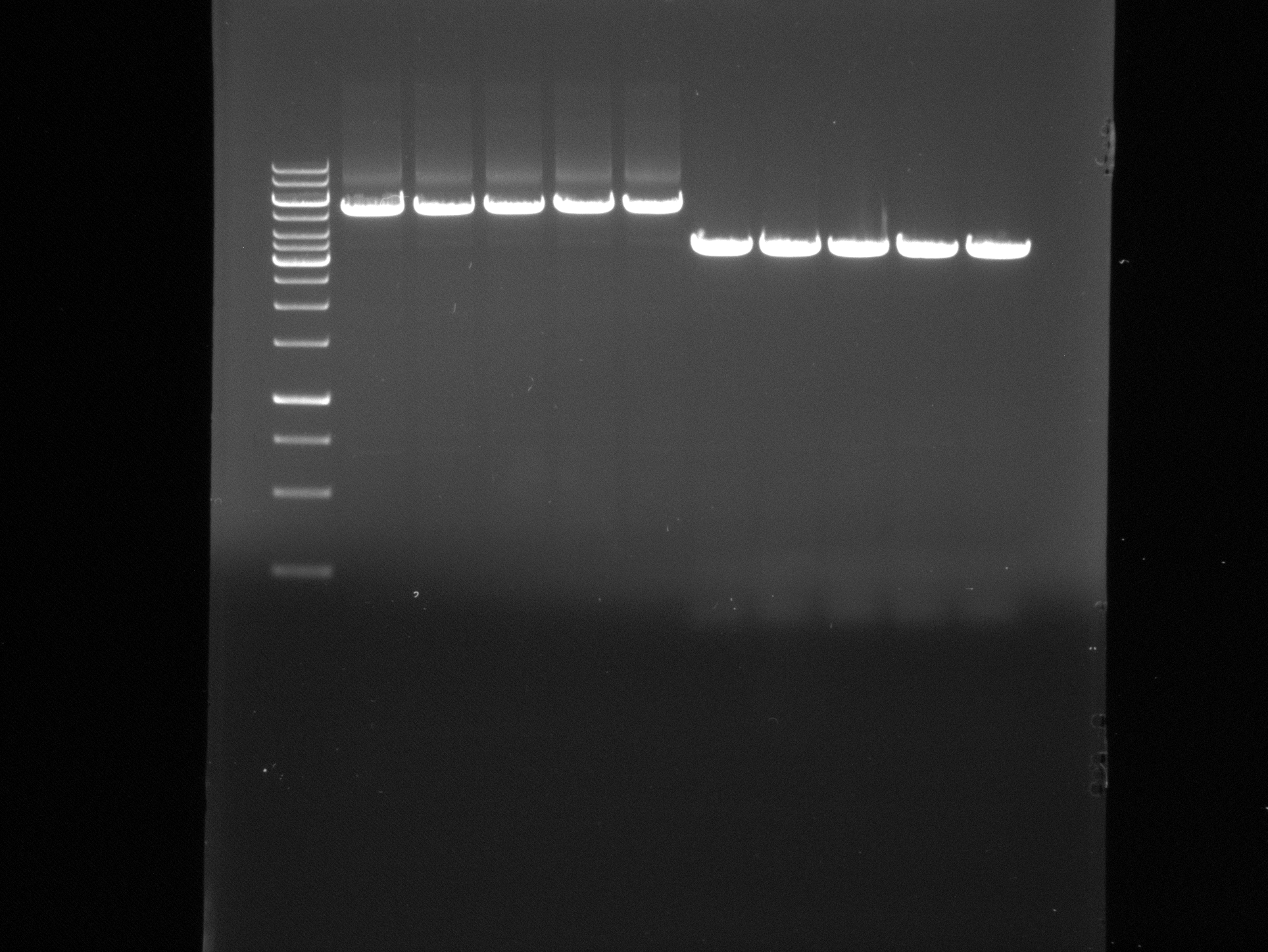

Supplement: Source data 1. [file elife-77095-data1.zip › source_suppfig1/Fig. S1 supplement-source1.tif]

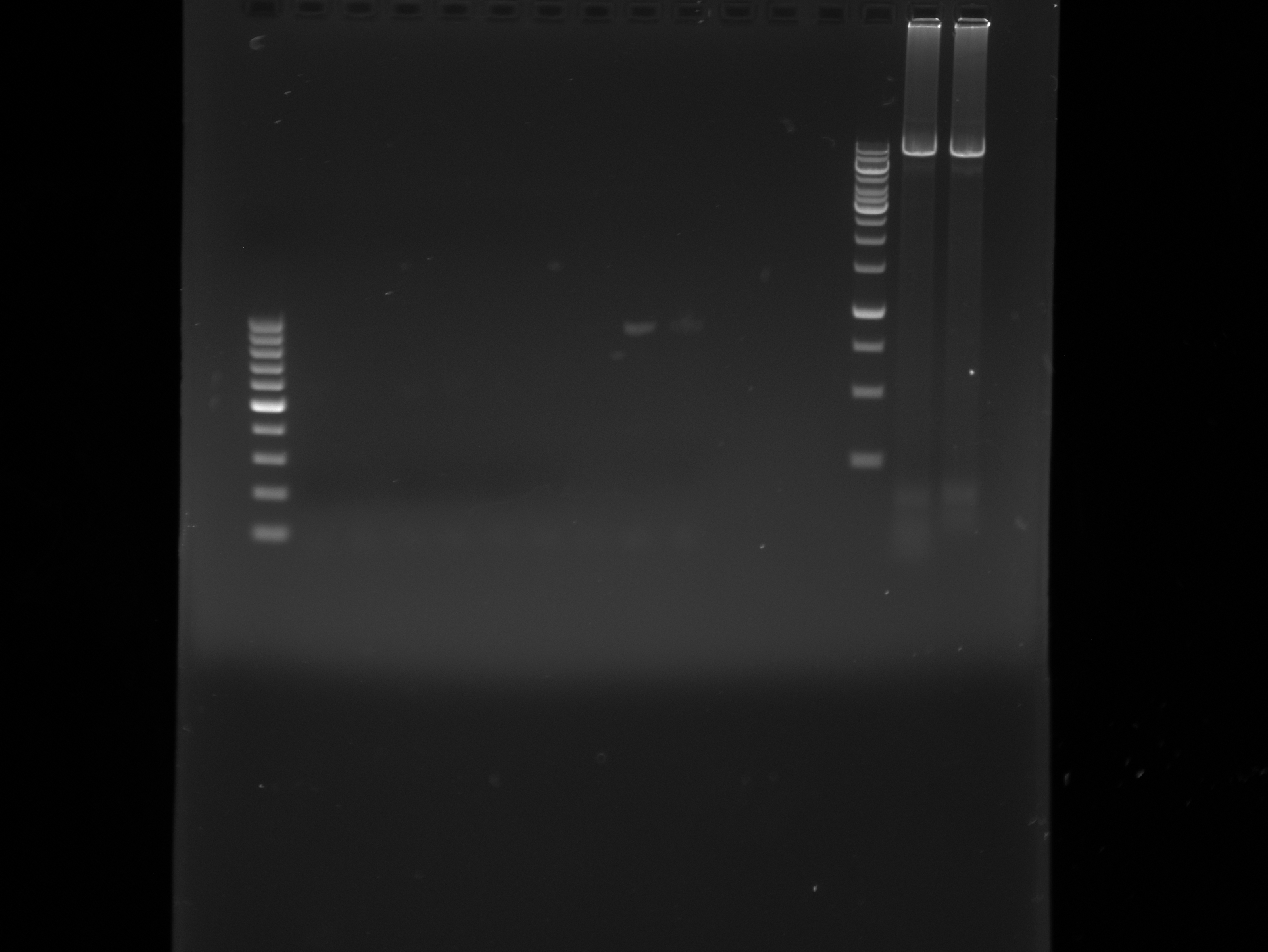

Supplement: Source data 1. [file elife-77095-data1.zip › source_suppfig1/Fig. S1 supplement-source2.tif]

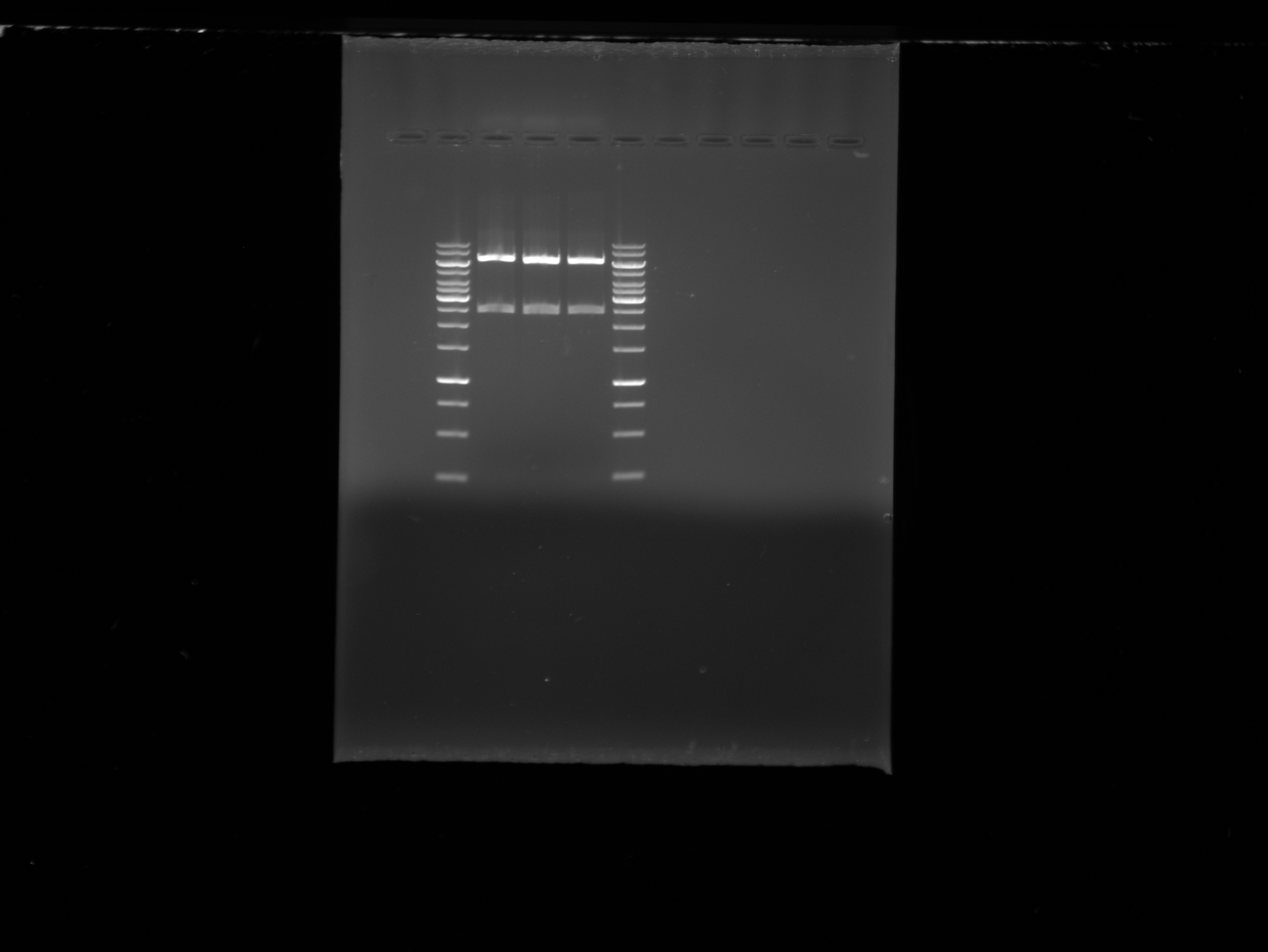

Supplement: Source data 1. [file elife-77095-data1.zip › source_suppfig1/Fig. S1 supplement-source3.tif]

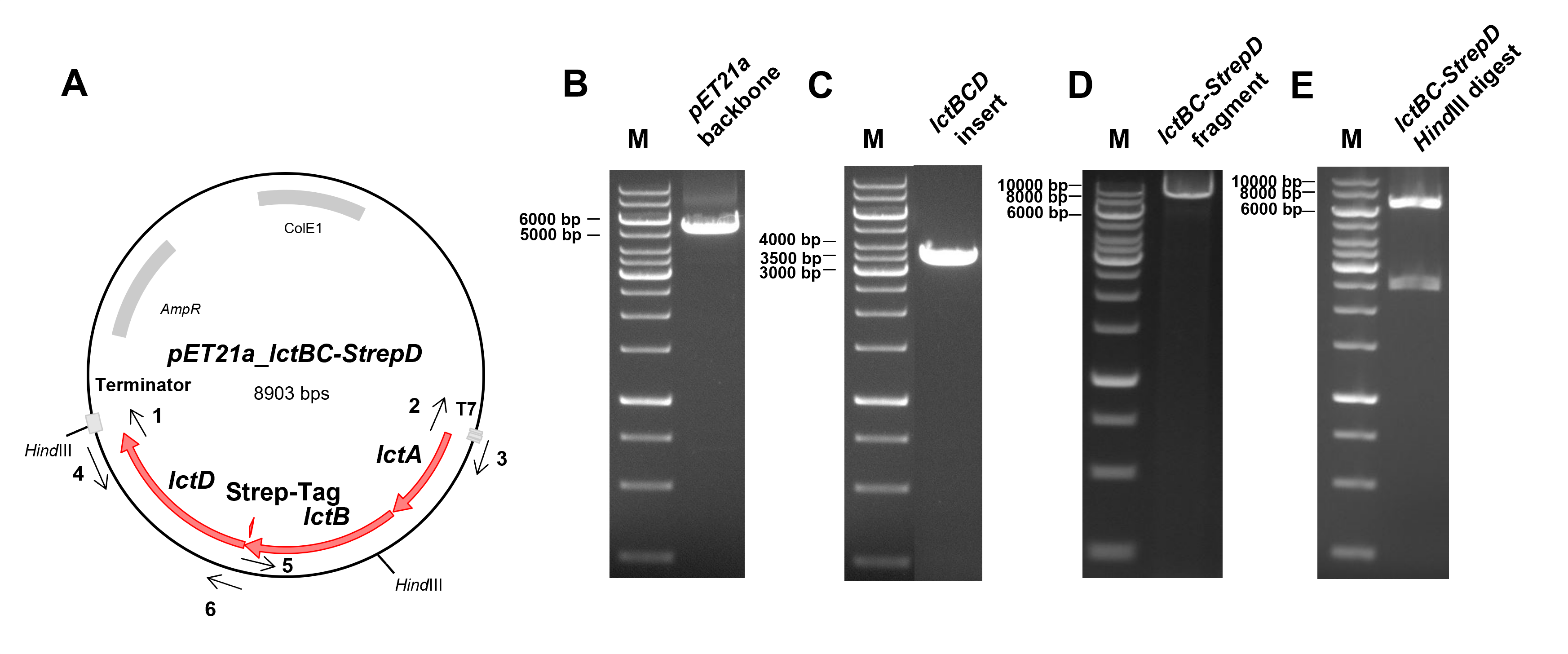

Supplement: Source data 1. [file elife-77095-data1.zip › source_suppfig1/Fig. S1 supplement.tif]
