# Supplementary material for: Structure-based electron-confurcation mechanism of the Ldh-EtfAB complex
Source: eLife. 2022 Jun 24;11:e77095. doi: 10.7554/eLife.77095 (PMC9232219; doi:10.7554/eLife.77095)
Supplement: Figure 6—source data 1. — WP_032489501.1, p-cresol methylhydroxylase of Pseudomonas putida; P56216.1, vanillyl-alcohol oxidase of Penicillium simplicissimum; WP_142969782.1, membrane-bound D-lactate dehydrogenase of Cronobacter sakazakii; WP_000097403.1, Escherichia. coli; WP_042389759.1, Pseudescherichia vulneris; WP_012062089.1 (highlighted in yellow); bifurcating lactate dehydrogenase of Alkaliphilus metalliredigens; WP_125071830.1, Lactiplantibacillus garii; WP_020224474.1, Holdemania massiliensis; WP_013278754.1, Acetohalobium arabaticum; WP_196599230.1, Pectinatus frisingensis; WP_113919857.1, Alkalibaculum bacchi; WP_014355267.1, A. woodii; WP_013387436.1, Ilyobacter polytropus; WP_012103837.1, Clostridium kluyveri; WP_195514995.1, Paraclostridium bifermentans. The postulated acid catalyst His423 is marked in red, the residues ligating the metal ion in green and Glu39 and Glu137 in cyan. [file elife-77095-fig6-data1.docx]

WP_032489501.1 ---MSEQNNAVLPKGVTQGEFNKAVQKFRALLGDDNVLVE-------SD--QLVPYNKIM 48

P56216.1 MSKTQEFRPLTLPPKLSLSDFNEFIQDIIRIVGSENVEVI-------SSKDQIVD-GSYM 52

WP_142969782.1 MILT-----------PTS-GNHEVIAELTRITGASH-LLTDPSKTARYRK---------- 37

WP_000097403.1 MSSM-----------TTT-DNKAFLNELARLVGSSH-LLTDPAKTARYRK---------- 37

WP_042389759.1 MSAV-----------SLQ-NNTLFLSELARLVGHAQ-LLTDPAKTARYRK---------- 37

WP_012103837.1 MNTD-----------LIKNSPEKVVENLKAIV------------GEDWVTSDLSKMQRYL 37

WP_125071830.1 ---M-----------ITTDQTTALYHQLTANF-DASAIQFGDQITADWGHDELGTVA--- 42

WP_020224474.1 ---------------MFRKLETKDIEALSAIV-GRQYVSVSDEIHPDYTHDEMTHYG--- 41

WP_013278754.1 ---M-----------SYNKVTEEDIKCLEEIL-DSDRVTIKENINEDYAHDELAELK--- 42

WP_196599230.1 --MK-----------PYKQVDEKDLNNIAEFI-DRDRLLWAKEVNEEYSHDELSAGQ--- 43

WP_113919857.1 ---M-----------QYEAIDQNDVNYLLSIVGEEGLFVGENNIHDDYSGDELAGIE--- 43

WP_014355267.1 ---M-----------NYKKVEASDIAAIKELI-PAERVFVGTEIGEDFSHDELGSIH--- 42

WP_013387436.1 ---M-----------NYKNVNIEDYENIAEILGNKERVLWEDGISEDYSHDELGGIS--- 43

WP_012062089.1 ---M-----------SYKKVDEKDVA-FFKSICGEERVFFGKEINEDFGRDELAEER--- 42

WP_195514995.1 ---M-----------EYKEFSVEDYKNILDLIGDNDRVLYGENISEDYSRDELGSVR--- 43

:

WP_032489501.1 MPV---------ENAAHAPSAAVTATTVEQVQGVVKICNEHKIPIWTISTGRNFGYGSAA 99

P56216.1 KPTHTHDPHHVMDQDYFLASAIVAPRNVADVQSIVGLANKFSFPLWPISIGRNSGYGGAA 112

WP_142969782.1 ----------GFRSGQGEALAVVFPGTLLELWRVLEVCVAADKIILMQAANT--GLTEGS 85

WP_000097403.1 ----------GFRSGQGDALAVVFPGSLLELWRVLKACVTADKIILMQAANT--GLTEGS 85

WP_042389759.1 ----------GFRSGEGKALAVVFPETLLQLWHVLNLCVRNDKIILMQAANT--GLTEGS 85

WP_012103837.1 YD----ETESLLRPEACKDCVVVKPASPEEISKILKYANKELLPVVVRGGGT--GVVAGA 91

WP_125071830.1 ----------------HLPDIVVTPKTTAEVATLAKLASAAKVPMTTRGSGT--GLVGGC 84

WP_020224474.1 ---------------CFDPELVIQPGCTEEVSQILAYANQENLPVTTRGAGT--GLCGGC 84

WP_013278754.1 ----------------VYPEVMVEPETTEEVSEIMKLASERNIPVTPRGTGT--GLCGGA 84

WP_196599230.1 ----------------YYPDIVARVVSTEEVAKLMRYASQNNIAITPRGAGT--GLVGAS 85

WP_113919857.1 ----------------RYPEVLVYPKNTQEISKILKYANENRIPVTSRGQGT--GLVGGS 85

WP_014355267.1 ----------------SYPEVLIKVTSTEEVSKIMKYAYEHNIPVVVRGSGT--GLVGAC 84

WP_013387436.1 ----------------RKPDILVKALDSSEISKVVKYAYEHSIPIVARGSGT--GLVGAS 85

WP_012062089.1 ----------------VMPEVLVEPSSTEEVSGIMRYANENNIPVTPRGQGT--GLVGGA 84

WP_195514995.1 ----------------VMPNVVVQALSAEEVSKVMKYAYNNNIPVTPRGSGT--GLVGAA 85

:: : . : . . * ..

WP_032489501.1 PVQRG-----QVILDLK-KMNKIIKIDPEMCYALVEPGVTFGQMYDYIQENNLPVMLSFS 153

P56216.1 PRVSG-----SVVLDMGKNMNRVLEVNVEGAYCVVEPGVTYHDLHNYLEANNLRDKLWLD 167

WP_142969782.1 TPNGNDYDRDIIIISTL-RLDKLHLLDNGEQ-ILAYPGTTLWQLERALKP------L-GR 136

WP_000097403.1 TPNGNDYDRDVVIISTL-RLDKLHVLGKGEQ-VLAYPGTTLYSLEKALKP------L-GR 136

WP_042389759.1 TPSGNDYDRDIVIISTL-RLDRLQLIDEGKQ-VLAYPGTTLYALEKALKP------L-GR 136

WP_012103837.1 IPTQP-----SIILSIE-RLNKVVEFDEKNIMITMEAGATLAQLLEVLSKNGK---L-FF 141

WP_125071830.1 VPTEG-----GLLIDMS-AMDQIIALDENNLSLTVQTGAQLKSVADYAAE-KG---F-LY 133

WP_020224474.1 VATQG-----GIVMSMM-RMNQILEIDEETMNAVIQPGVLLMEIIEEAAD-HN---L-LY 133

WP_013278754.1 VAMEG-----GILLLTT-AMDEIIEIDEENLTAKVQPGVILMSFAEKVND-LG---F-MY 133

WP_196599230.1 VAIEH-----GIMLDTT-LMNHFLELDEQNLTLTLEPGVLLMELADYVEK-RG---Y-FY 134

WP_113919857.1 VALYG-----GIMLSTE-KMNNILELDEENLTVTVEPGVLLMDLTAFVES-HD---L-FY 134

WP_014355267.1 VPLFG-----GIMLETT-LMNNILELDTENLTVTVEPGVLLMELSKFVEE-ND---L-FY 133

WP_013387436.1 VPIHG-----GIMIETT-QMNKILELDEDNLTLTVEPGVLLMEIGKYVED-RD---F-FY 134

WP_012062089.1 VAIHG-----GIMLNMS-RMNQILEIDEDNLTLTVEPGVLLMEISKFVEE-HD---L-FY 133

WP_195514995.1 VPIKA-----GIVIDLS-KMNKILELDEENLTLTVEPGVLLMEIGKYVEE-FD---L-FY 134

::: ::.. .. *. .

WP_032489501.1 APSAI------AG-PVGNTM-----DRGVGYTPYGEHFMMQCGMEVVLANGDVYRTGMGG 201

P56216.1 VPDLG------GGSVLGNAV-----ERGVGYTPYGDHWMMHSGMEVVLANGELLRTGMGA 216

WP_142969782.1 EPHSVIGSSCIGASVMGGICNNSGGALVHRGPAYTEMALFAQ----------IDDTGRLR 186

WP_000097403.1 EPHSVIGSSCIGASVIGGICNNSGGSLVQRGPAYTEMSLFAR----------INEDGKLT 186

WP_042389759.1 EPHSVIGSSCIGASVIGGVCNNSGGSLVQRGPAYTEMSLFAR----------IDEQGQLR 186

WP_012103837.1 PVHPG----DEGAQVGGMVAANAGGTRAV---KHGIMRNHVKALEVVLATGEIVTLGGKL 194

WP_125071830.1 APDPG----EKTATIGGNIATNAGGMRAV---KYGVTRESVRALTVVTVDGQVLHLGGQI 186

WP_020224474.1 APDPG----EKSASVGGNVMTNAGGMRAV---KYGVTRDYVRGLEVVLADGSVLMLGGKT 186

WP_013278754.1 PPDPG----EKSATLGGNVLTNAGGMRAV---KYGVTRDYVLGMEIVLPNGEVINTGGKV 186

WP_196599230.1 PPDPG----EKSATIGGNISTNAGGMRAV---KFGVTRDYIRGLEIVLADGTVMTVGGKA 187

WP_113919857.1 PPDPG----EKSASIGGNVSTNAGGMRAV---KYGVTRDFVRGIEFVMPDGTVMNFGGKI 187

WP_014355267.1 PPDPG----EKSATIAGNISTNAGGMRAV---KYGVTRDYVRGLTVVLANGEIIELGGKI 186

WP_013387436.1 PPDPG----EKSATIGGNISTNAGGMRAV---KYGVTRDYVRGLEVVLPNGDIMEMGGKV 187

WP_012062089.1 PPDPG----EKSATIAGNINTNAGGMRAV---KYGVTRDFVRGLEVVLPNGEVLEIGGKV 186

WP_195514995.1 PPDPG----EKTATIGGNISTNAGGMRAV---KYGVTRDYVRGLEVVLPNGEIINVGGKV 187

. * . : *

WP_032489501.1 VPGSN-------------------------------------------TWQIFKWGYGPT 218

P56216.1 LPDPKRPETM------GLKPEDQP---------------------WSKIAHLFPYGFGPY 249

WP_142969782.1 LVNHLGIDLGQTPEQILGRLDDDRIDPAAVRHDERQASDRHYIERVRDIEADSPARYNAN 246

WP_000097403.1 LVNHLGIDLGETPEQILSKLDDDRIKDDDVRHDGRHAHDYDYVHRVRDIEADTPARYNAD 246

WP_042389759.1 LVNHLGIDLGETPEQILSKLDDQRVNDADVRHDGRHAHDSDYVERVRDIEADTPARYNAD 246

WP_012103837.1 LKNNMGYDLL-------------------------------------------------- 204

WP_125071830.1 VKNSSGFDLK-------------------------------------------------- 196

WP_020224474.1 SKNSSGYSLK-------------------------------------------------- 196

WP_013278754.1 VKNSSGYSIK-------------------------------------------------- 196

WP_196599230.1 VKNSSGYDLK-------------------------------------------------- 197

WP_113919857.1 VKNSSGYSLK-------------------------------------------------- 197

WP_014355267.1 VKNSSGYSLK-------------------------------------------------- 196

WP_013387436.1 VKNSAGYSLK-------------------------------------------------- 197

WP_012062089.1 VKNSSGYSLK-------------------------------------------------- 196

WP_195514995.1 VKNSSGYALK-------------------------------------------------- 197

.

WP_032489501.1 LDGMF----TQANYGICTKMGFWLMPKPPVFKPFEVIFEDEADIVEIVDALRPLRMSNTI 274

P56216.1 IDGLF----SQSNMGIVTKIGIWLMPNPRGYQSYLITLPKDGDLKQAVDIIRPLRLGMAL 305

WP_142969782.1 PERLFEASGCAGKLAVFA-VRLDTFEAEKRQEVFYIGTNQPDVLTHIRRHI--LANFQSL 303

WP_000097403.1 PDRLFESSGCAGKLAVFA-VRLDTFEAEKNQQVFYIGTNQPEVLTEIRRHI--LANFENL 303

WP_042389759.1 PDRLFESSGCAGKLAVFA-VRLDTFAAAKRQQVFYIGTNQPAVLTEIRRHM--LANFDNL 303

WP_012103837.1 -QLMI---GGEGTLGVITKVTLRLYAASKYNGTLLVSFNSQREACDAVP-E--ILQEGIT 257

WP_125071830.1 -DLYI---GSEGTLGIVTEAVLKISPLPKYSTGLLIPFDDFDSALTTVP-K--ILQSGIT 249

WP_020224474.1 -DLIV---GSEGTLAVVTKIIMKLLPKPRAMTSLLIPFNTLGQALTLVP-K--IMRLPAV 249

WP_013278754.1 -DLMV---SSEGTLGIVTEITLKLIPLPKKQLTLLIPFDSLDEAIDTVP-E--IINAKIV 249

WP_196599230.1 -DVII---GSEGTLAVITKAILKLLPLPQKTVSLLVPFPTLAQAIGAVP-P--IIKSKAI 250

WP_113919857.1 -DFVV---GSEGTLGVISKLILKLLPLPKHKLSLLVPFKDLSSAIKTVP-E--IIKSKSI 250

WP_014355267.1 -DLVI---GSEGTLCVITKAILKLLPLPKMTLSLLIPFENISDAAGIVP-K--IIKSKAI 249

WP_013387436.1 -DLVI---GSEGTLGIITKAVLKLLPLPKYSISLLIPFGNIDNAIDAVP-T--IIRSKAI 250

WP_012062089.1 -DLIV---GSEGTLGIVTKAILKLLPLPKKAISLLVPFPNLELAIETVP-K--IIKSKST 249

WP_195514995.1 -DLIV---GSEGTLGIVTKAVLRLLPLPKKAISLLIPFDTLDNAIETVP-K--IIKSKSI 250

: . .. : : : : :

WP_032489501.1 PNSVV--IASTLW-----------EAGSAHLTRAQYTTEPGHTPDSVIKQMQKD---TGM 318

P56216.1 QNVPT--IRHILL-----------DAAVLG-DKRSYSSRTEPLSDEELDKIAKQ---LNL 348

WP_142969782.1 PVAGEYMHRDIYDIAERYGKDTFLMINKLGTDKMPFFFTAKGRTDAWLEKVPFVKPHFTD 363

WP_000097403.1 PVAGEYMHRDIYDIAEKYGKDTFLMIDKLGTDKMPFFFNLKGRTDAMLEKVKFFRPHFTD 363

WP_042389759.1 PVAAEYMHRDIYDIAERYGKDTFLMIDKLGTDKMPMLFTLKGRADAMLEKVTLFKPHFTD 363

WP_012103837.1 PLAIEYMDRVICEESAKQLGTKWPAVKGS----VDLMFILDYASEEDLYS---------- 303

WP_125071830.1 PTAVEFFEADTVKYWEAFKKQDFPQAGAA----AYLLMTLDGQHEEVVEQ---------- 295

WP_020224474.1 ATTIEFMEKEVIQDAQDYLGKDFPNKEFN----AYLIVSYSANTPEEMQA---------- 295

WP_013278754.1 PTGIEFMEREVLLAATDYLGKGFPETSAP----AYLLLTFYGNDNEELEK---------- 295

WP_196599230.1 PTAIEFMEREVIIDAEEYLGRKFPDNQAD----AYLLLKFDGNTLEEIAS---------- 296

WP_113919857.1 PTAIEFMQGKVILASEEFLGKKFPDNSSD----AYLLLQFDGNSKEEIEM---------- 296

WP_014355267.1 PTAIEFMERQTILFAEDFLGKKFPDSSSN----AYILLTFDGNTKEQVEA---------- 295

WP_013387436.1 PTAVEFMQKEVIYSAEEFLGKTFPDKSSD----AYLLLTFDGNSKEQVEK---------- 296

WP_012062089.1 PTAVEFMVRDTIIAAEEFLGKKFPDNSAD----AYLLLTFDGNSKEEVER---------- 295

WP_195514995.1 PTAIEFMQRAAIVAAEEFLKKPFPDNSSD----AYLLLTFDGNSKEEIER---------- 296

:

WP_032489501.1 GAW-NLYAALYGTQEQVDVNWKIVT---------------------DVFKKLGKGRIVTQ 356

P56216.1 GRW-NFYGALYGPEPIRRVLWETIK---------------------DAFSAIPGVKFYFP 386

WP_142969782.1 RVMQKLGGLFPGHLPPRMKSWRDKYEHHLLLKMAGDGIEEARSWLTSFFKEAEGDFFACT 423

WP_000097403.1 RAMQKFGHLFPSHLPPRMKNWRDKYEHHLLLKMAGDGVGEAKSWLVDYFKQAEGDFFVCT 423

WP_042389759.1 RLMQRVGSVFPGHLPPRMKSWRNKYEHHLLLKMAGDGIAEAQSWLKEYFTEAEGGFFACT 423

WP_012103837.1 -----------------------NSEKLVE----------------ICERHNSVDSIIAE 324

WP_125071830.1 -----------------------DYETLAE----------------LCLDNGATDAYVLD 316

WP_020224474.1 -----------------------MIHDCAE----------------LALQEGAEDVFISD 316

WP_013278754.1 -----------------------KYEAAAD----------------VCLDNDAYDVYIAN 316

WP_196599230.1 -----------------------YYDGVAQ----------------ICLEQGAADILIAD 317

WP_113919857.1 -----------------------NYEKVAD----------------ICLECGALDVLISD 317

WP_014355267.1 -----------------------EYETVAN----------------LCLAEGAKDVYIVD 316

WP_013387436.1 -----------------------DYEVVAD----------------LCLEIGATDVYIVD 317

WP_012062089.1 -----------------------AYEGVAH----------------ICLEAGALDVYISE 316

WP_195514995.1 -----------------------AYEGVAN----------------ICLESGALDVFISD 317

WP_032489501.1 EEAGDTQPFKYRAQLMSGVPN----LQEF----GLYNWRGGGGSMWFAPVSEARGSECKK 408

P56216.1 EDTPENSVLRVRDKTMQGIPT----YDEL----KWIDWLPNGAHLFFSPIAKVSGEDAMM 438

WP_142969782.1 AEEGSKAFLHRFAAAGAAVRYHAVHADEVEDILALDIALRRNDTEWFE------------ 471

WP_000097403.1 PEEGSKAFLHRFAAAGAAIRYQAVHSDEVEDILALDIALRRNDTEWYE------------ 471

WP_042389759.1 TEEGNKAFLHRFAAAGAAIRYQAVHADEVEEILALDIALRRNDTEWFE------------ 471

WP_012103837.1 TEKEQRHLLEIRSNAYGPYKNN--IADIM------DVAVPPSSVPDFFDDVK-------- 368

WP_125071830.1 TPELKETVWKARDAFLEAIQASTTTMDEA------DAVVPRDQTAEFVAFTH-------- 362

WP_020224474.1 TEERQSSIWDARGAFLEAIKNSTTQMDEC------DVVVDIDKVAEFCQFAR-------- 362

WP_013278754.1 TEERQDVIWDTRGALLEALKAV-SHLDEC------DVVVPRNRVAEFVKYTH-------- 361

WP_196599230.1 TEERAEAIWKARGAFLEAIKSSTTMMDEV------DVVVPRSMVNDFVEYIH-------- 363

WP_113919857.1 TDERHDSLWTARGAFLEAIKASTTQMDEC------DVVVPRNKVADFIIFTD-------- 363

WP_014355267.1 TVERKDSVWSARGAFLEAIKASTTEMDEC------DVVVPRNRIAEFIEFTH-------- 362

WP_013387436.1 TEERKESVWSARGAFLEAIKASTDEMDEC------DVVVPRNCVSEFIKYTK-------- 363

WP_012062089.1 TQERQEAIWSARGAFLEAIKASTTQIDEC------DVVVPRNHVAKFIRYTN-------- 362

WP_195514995.1 TEERQEAIWSARGAFLEAIKALTTEMDEV------DVVVPRNKVAEFVKFTH-------- 363

: . :

WP_032489501.1 QAAMAKRVLHKYGLDYVAEFIVAPRDMHHVIDVLYD-----RTNP---EETKRADACFNE 460

P56216.1 QYAVTKKRCQEAGLDFIGTFTVGMREMHHIVCIVFN-----KKDL---IQKRKVQWLMRT 490

WP_142969782.1 ------RLPPEIDSKL-----VHKLYYGHFFCHVFHQDYIVKKGV-------DVHALKEE 513

WP_000097403.1 ------HLPPEIDSQL-----VHKLYYGHFMCYVFHQDYIVKKGV-------DVHALKEQ 513

WP_042389759.1 ------TLPEAIASKL-----THKLYYGHFFCHVFHQDYIVKRGV-------DVHELKAQ 513

WP_012103837.1 ------RLTKEYDNKI-----V---SLGHIGDGNIH-NFIMGDNG---KLPANYEELKEA 410

WP_125071830.1 ------HLAEQQNVRI-----P---GFGHVGDGNLH-LYVCQDDYSDTEWPAKLKTVFEA 407

WP_020224474.1 ------DLSLQEDIRI-----R---SFGHAGDGNLH-IYVLKDALEEAVWQEKVTRCMDA 407

WP_013278754.1 ------KLEEKHDLRI-----R---SFGHAGDGNLH-IYTLKDDLDEETWHERNKAVMDD 406

WP_196599230.1 ------DLQKEIGVRI-----K---CFGHAGDGNLH-AYILKDQLNDEQWQKVLKATMEK 408

WP_113919857.1 ------QCQKECDIRI-----S---SFGHAGDGNLH-VYLLRDEMDEETWNKKAAAVFER 408

WP_014355267.1 ------DLAKEMDVRI-----P---SFGHAGDGNLH-IYVCRDELCQADWEAKLAEAMDR 407

WP_013387436.1 ------ELEKEFDIRI-----P---SFGHAGDGNLH-IYICRDGLEQEKWEEKLSAVFKK 408

WP_012062089.1 ------ELQKEFDIRI-----R---SFGHAGDGNLH-VYILRDELTQEVWKEKLNAVMDC 407

WP_195514995.1 ------EVEESQDIRI-----K---SFGHAGDGNLH-IYILRDELNSEEWNNKLNNVMEI 408

* .

WP_032489501.1 LL---DEFEKEGYAVYRVNTRFQDRVAQSYGPVK---RKLEHAIKRAVDPNNILAPGRSG 514

P56216.1 LI---DDCAANGWGEYRTHLAFMDQIMETYNWNNSSFLRFNEVLKNAVDPNGIIAPGKSG 547

WP_142969782.1 MLALLRERGAQYPAEHNVGHLYEA-------------PESLKQFYKANDPTNSMNPGIGK 560

WP_000097403.1 MLELLQQRGAQYPAEHNVGHLYKA-------------PETLQKFYRENDPTNSMNPGIGK 560

WP_042389759.1 MLALLHERGAQYPAEHNVGHLYEA-------------AESLKRNYRELDPTNSMNPGIGK 560

WP_012103837.1 IYKTAIKYGGTITAEHGTGKLRKKHMPLQFSKRE---IEIMEGIKKVFDPNGILNQGDMV 467

WP_125071830.1 LYAKASELGGKVSGEHGIGYVKRPYLQSTSDSAE---MATMRRVKAALDPDNLLNPDKIV 464

WP_020224474.1 LYEKAAAMKGQVSGEHGIGHAKREYLRQSLQPAQ---IELMRRIKAAFDPNGILNPGKVV 464

WP_013278754.1 MYAKAREMKGQVSGEHGIAYAKKEYLHEDIGKTQ---VELMKGIKEVFDPQNILNPGKTV 463

WP_196599230.1 MYTKARSLKGNVSGEHGIGYAKKGYLKESMPDKA---IAIMQGIKQVFDPLNILNPHKVC 465

WP_113919857.1 MYQKAFEMGGAVSGEHGIGFAKLPYLEEEFGEKG---MELFRRIKLAFDPNEIMNPGKLG 465

WP_014355267.1 MYAKALTFEGLVSGEHGIGYAKRKYLLNDFGTEH---LALMAGIKQTFDPKNLLNPKKVC 464

WP_013387436.1 MYTRSEELGGLVSGEHGIGYAKKNYMFDQYCENN---IEIMRGIKQVFDPKNILNPGKVC 465

WP_012062089.1 MYSRAKELNGQVSGEHGIGIAKKAYLSESLGATP---LAIMAGIKASFDPRGILNPGKVC 464

WP_195514995.1 LYKKSRELNGQVSGEHGIGFAKKPFLNESLPKES---IEIMKGIKLAFDPKNILNPGKVC 465

: . : ** :

WP_032489501.1 IDLNNDF---------------- 521

P56216.1 VWPSQYSHVTWKL---------- 560

WP_142969782.1 TSKQKYWGEPDGIAQTPGEQTAR 583

WP_000097403.1 TSKRKNWQEVE------------ 571

WP_042389759.1 TSKRKYWGEGADPSQS------- 576

WP_012103837.1 D---------------------- 468

WP_125071830.1 ----------------------- 464

WP_020224474.1 E---------------------- 465

WP_013278754.1 ----------------------- 463

WP_196599230.1 QD--------------------- 467

WP_113919857.1 SNYRVSLY--------------- 473

WP_014355267.1 QM--------------------- 466

WP_013387436.1 Q---------------------- 466

WP_012062089.1 ----------------------- 464

WP_195514995.1 Q---------------------- 466

**Figure 6 – figure supplement 1.** Sequence alignment of Ldh. WP_032489501.1, p-cresol methylhydroxylase of *Pseudomonas putida*; P56216.1, vanillyl-alcohol oxidase of *Penicillium simplicissimum*; WP_142969782.1, membrane-bound D-lactate dehydrogenase of *Cronobacter sakazakii*; WP_000097403.1, *Escherichia coli*; WP_042389759.1, *Pseudescherichia vulneris*; WP_012062089.1 (highlighted in yellow)*;* bifurcating lactate dehydrogenase of *Alkaliphilus metalliredigens*; WP_125071830.1, *Lactiplantibacillus garii*; WP_020224474.1, *Holdemania massiliensis*; WP_013278754.1, *Acetohalobium arabaticum*; WP_196599230.1, *Pectinatus frisingensis*; WP_113919857.1, *Alkalibaculum bacchi*; WP_014355267.1, *A. woodii*; WP_013387436.1, *Ilyobacter polytropus*; WP_012103837.1, *Clostridium kluyveri*; WP_195514995.1, *Paraclostridium bifermentans*. The postulated acid catalyst His423 is marked in red, the residues ligating the metal ion in green and Glu39 and Glu137 in cyan.
