# Supplementary material for: Structure-based electron-confurcation mechanism of the Ldh-EtfAB complex
Source: eLife. 2022 Jun 24;11:e77095. doi: 10.7554/eLife.77095 (PMC9232219; doi:10.7554/eLife.77095)
Supplement: Supplementary file 1. — II. Cloning of pET21a_lctBC-StrepD. [file elife-77095-supp1.docx]

**Supplementary File 1**

**1.1 Table of corresponding primers used.**

| **Primer** | **Sequence (5’→ 3’)** |
| --- | --- |
| lctBCD_pET21a_for | tttaagaaggagatatacatATGAAAATACTGGTATGTATCAAAC |
| lctBCD_pET21a_rev | acggagctcgaattcggatcCTACATCTGACAGACTTTTTTC |
| pet21a_for | GATCCGAATTCGAGCTCC |
| pet21a_rev | ATGTATATCTCCTTCTTAAAGTTAAAC |
| lctBCD_pMTL84211_fwd | ACCGCTATTGATGTTGCTCCGACGGAAGCCATTCTTCAGGAAGG |
| lctBCD_pMTL84211_rev | TCCAGCGTTTGTAGCTGCGTCACCAATAACCGCTATTTTATTTTCAATCGTGATG |
| pMTL84211_fwd | AGTTAGGGAATGTTACTTTGTAGTG |
| pMTL84211_rev | GGATCCGTCCTCCCTTTAAATTTAAC |
| ∆Fe/S_for | gccaaaatggccaaaatggccCTTAAAAAAGGACCTGAAGGGG |
| ∆Fe/S_rev | AGCTGCCGTGACTTCCAG |
| ∆Fe/S-arm_for | GACAAAAGCTTATATCGG |
| ∆Fe/S-arm_rev | CATGATTTTTTCCTTTCTAGCC |
| ΔR205_for | AGTTCAGATTgcaCCAGCTTTTG |
| ΔR205_rev | AAATCGGTGTTTTCTTTCATTTC |
| ΔD189_for | ACTTACCGCTggaTGTACCATTTTGGAAATG |
| ΔD189_rev | CCGGTTCGATAACGAGCC |
| Δβ-FAD_for | aggagcaggagcaGAAATGGCTGAATTTTTGGG |
| Δβ-FAD_rev | gctcctgctcctgcGGTGGTTTGTTTACCGCA |
| ΔNAD_for | aggcgcaGCCGATGTGGTAGCGACC |
| ΔNAD_rev | gcttttgcATCCGATAATAAACAACCCTCATCAG |
| ΔSPT_for | gcaggagcaCAGGTGGAACGAATTTTCC |
| ΔSPT_rev | TCCGCTCAGTCCATATTTTTTTTC |
| Strep-Tag_for | ccacagttcgagaagTAAGATGAATTATAAAAAAGTGGAAGC |
| Strep-Tag_rev | gtgggaccatgctgaAACGACCATCCTTTCCGG |

Plasmids generated with exchanges in EtfA are pET21a_lctBCD[∆Fe/S](C41A, C44A, C47A), pET21a_lctBCD[∆Fe/S-arm](∆2A-65I), pMTL84211_lctBCD[∆Fe/S](C41A, C44A, C47A), pMTL84211_lctBCD[∆Fe/S-arm](∆2A-65I), pET21a_lctBCD[ΔR205](R205A) and pET21a_lctBCD[ΔD189](D189A). Plasmids generated with exchanges in EtfB are pET21a_lctBCD[Δb-FAD](D122A, D124A, T125G, Q127G, V128A, P130A), pET21a_lctBCD[ΔNAD](R87A, F89A, G91A) and pET21a_lctBCD[ΔSPT](S223A, P224G, T225A).

**
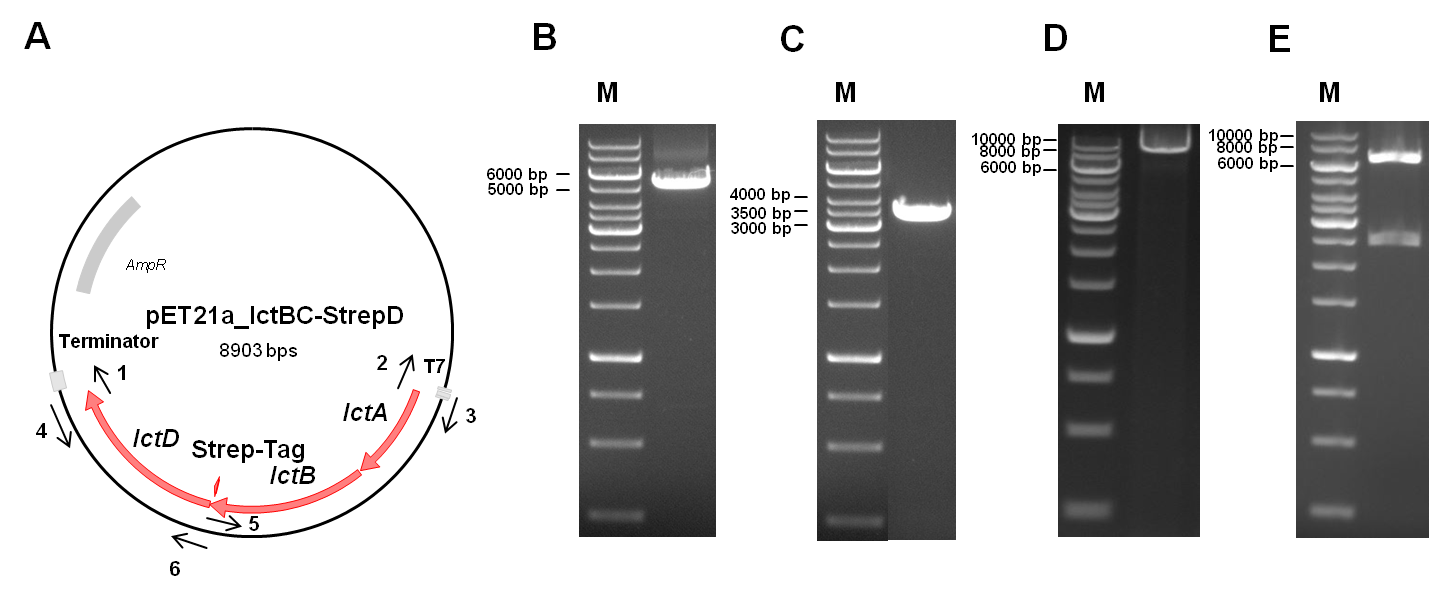
**

**1.2 Cloning of *pET21a_lctBC-StrepD*.** For the production of Ldh-EtfAB-Strep in *E. coli* the construct *pET21a_lctBC-StrepD* was cloned (**A**) Therefore, *pET21a* backbone, including a T7-promotor, was amplified using corresponding primers pET21a_for (1) and pET21a_rev (2) via PCR (size: 5406 bp) (**B**) *LctBCD* was amplified from genomic DNA of *A. woodii* via PCR, using lctBCD_pET21a_for (3) and lctBCD_pET21a_rev (4) primers (size: 3537 bp) (**C**) Amplified *lctBCD* and *pET21a* backbone were fused via Gibson Assembly and transformed in *E. coli* HB101. Afterwards, plasmids where isolated and a sequence encoding for a Strep-tag was introduced at the 3’-end of the gene *lctC* by using corresponding primers Strep-Tag_for (5) and Strep-Tag_rev (6) (size: 8903 bp) (**D**) The resulting *pET21a_lctBC-StrepD* was digested with *Hind*III (E). The resulting sizes were 6393 bp and 2510 bp. M, Gene Ruler 1 kb DNA ladder.

**Source data 1.** Source data for Supplementary File 1-II
